# Measuring problematic smartphone use among students using the Smartphone Addiction Scale

**DOI:** 10.4102/safp.v67i1.6120

**Published:** 2025-05-30

**Authors:** Nicky Mostert

**Affiliations:** 1 Department of Software Engineering, Faculty of Engineering, Built Environment and Technology, Nelson Mandela University, Gqeberha, South Africa

**Keywords:** problematic smartphone use, smartphone addiction, primary care, mental health, Smartphone Addiction Scale, university students

## Abstract

**Background:**

Problematic Smartphone Use (PSU) is a growing concern, particularly among university students, due to its potential negative impacts on mental health, academic performance, and daily functioning. Characterized by compulsive smartphone use, PSU is linked to anxiety, depression, and sleep disturbances. Understanding PSU in university settings is essential for creating effective interventions. Additionally, primary care settings can play a key role in identifying and managing PSU to support overall well-being.

**Methods:**

This cross-sectional study involved 867 students from Nelson Mandela University, South Africa. Participants completed the Smartphone Addiction Scale – Short Version (SAS-SV) to assess PSU levels. The study explored associations between PSU, daily smartphone usage time, and gender using descriptive statistics and correlation analyses.

**Results:**

The findings revealed that 55% of the students exhibited PSU. A significant correlation was observed between increased daily smartphone usage and higher PSU levels. However, no significant gender differences were found. These results highlight the widespread nature of PSU among university students and its strong association with smartphone usage patterns.

**Conclusion:**

The study underscores the importance of addressing PSU in primary healthcare settings, where early detection and intervention can prevent further psychological and social consequences. Primary care providers can offer counselling, screen for PSU, and guide students towards healthier smartphone habits.

**Contribution:**

This study provides valuable insights into the prevalence and impact of PSU among university students. It emphasizes the vital role of primary care in addressing this emerging health issue to improve students’ mental health and overall quality of life.

## Introduction

For many individuals, smartphones have become an essential part of daily life. The wide range of features offered by smartphones has made them an important tool in modern society. However, despite the advantages, the increase in screen time has raised concerns about its impact on users’ mental health.^1^ Problematic Smartphone Use (PSU), often referred to as smartphone addiction, is defined as a compulsive pattern of smartphone use, marked by a persistent and overwhelming desire to use the device, which individuals struggle to control.^2^ This excessive use can lead to negative consequences, hindering the user’s ability to function effectively in daily activities.^3^ Problematic Smartphone Use occurs when individuals find it difficult to manage their smartphone use, leading to disruptions in their personal, social or professional lives. Given the crucial role smartphones play in students’ lives, addressing PSU is not only a matter of personal concern but also a significant public health issue.

Several studies have explored the causes and effects of PSU on university students, particularly its negative impact on academic performance.^4,5,6,7,8,9^ For a university to implement effective strategies to reduce the risk of PSU among students, it must first understand the extent of PSU within its student body. A study such as this represents a first step in assessing PSU levels among students at a university in South Africa to understand the extent of PSU within its student body. In addition to measuring PSU, two hypotheses were also formulated:

**H1:** There are significant differences in university students’ PSU levels based on gender.

**H2:** There are significant differences in university students’ PSU levels based on the amount of time spent on their phones each day.

In primary healthcare settings, identifying and addressing PSU among university students can have a profound impact on overall mental health. With students often navigating high levels of stress, academic pressures and social demands, excessive smartphone use can exacerbate anxiety and depression, and can even contribute to more severe mental health disorders. Therefore, measuring the prevalence of PSU is essential in understanding its scope and devising appropriate interventions that could mitigate its adverse effects. This study, conducted at Nelson Mandela University, provides important insights into PSU among students in South Africa, highlighting its prevalence, the role of daily smartphone use, and the lack of gender differences in addiction patterns. These findings are critical in the development of primary healthcare strategies that address PSU not only in academic environments but also across healthcare systems, aiming to improve students’ academic success and mental health outcomes.

By focusing on the relationship between smartphone usage and mental health, this study offers a foundation for healthcare professionals to develop targeted interventions that promote healthier smartphone habits. This is especially relevant in university settings, where students are at high risk of developing dependence on their devices. Addressing PSU within primary healthcare frameworks will help ensure that students receive the support they need to navigate the challenges posed by digital technology in a balanced and healthy way.

## Research methods and design

### Study design

This study employed a cross-sectional, descriptive design to explore the prevalence of PSU among university students at Nelson Mandela University. A quantitative approach was adopted, utilising an online survey to collect data on students’ smartphone usage patterns and associated behaviours. The design aimed to assess PSU levels and identify potential associations between PSU and factors such as gender and daily smartphone usage time. A descriptive analysis was used to provide an overview of the sample population’s characteristics and PSU symptoms.

### Participants

#### Setting

The study was conducted at Nelson Mandela University in South Africa, a higher education institution with a diverse student population. Data collection took place in April 2024, and the participants were enrolled students aged 18 years or older. The setting was chosen because of the prevalence of smartphone use among university students, as well as the emerging concerns about PSU and its impact on academic performance and mental health within the university environment.

#### Study population and sampling strategy

The study population consisted of students aged 18 years and older, enrolled at Nelson Mandela University. The total registered student population at the time of data collection was 31 311. A non-probability, convenience sampling strategy was employed, where students were invited to participate via an email sent to all registered students. The email included a link to an online questionnaire for voluntary participation.

To calculate the required sample size, a 95% confidence level, 5% margin of error and a 50% population proportion were applied, resulting in a minimum required sample size of 380 participants. However, 867 students participated, which exceeds the minimum requirement, ensuring adequate statistical power. The final sample was diverse in terms of gender, academic department and qualification level, allowing for a broad understanding of PSU patterns within the student population.

### Data collection

Data were collected using an online survey administered through a link provided to students via email. The survey included demographic questions (e.g. gender, age, academic department and qualification level) and the Smartphone Addiction Scale – Short Version (SAS-SV) to assess PSU. The SAS-SV comprises 10 items rated on a 6-point Likert scale, designed to measure symptoms associated with PSU, such as loss of control, disruption of academic or personal life, disregard for consequences, withdrawal, preoccupation and tolerance.

### Data analysis

Data analysis was conducted using descriptive and inferential statistics. Descriptive statistics were used to analyse demographic characteristics and participants’ responses to the SAS-SV items. The mean SAS-SV score was calculated to determine the overall level of PSU within the sample. Inferential statistics, including chi-square tests, were used to explore the relationships between PSU levels and independent variables such as gender and the amount of time spent on smartphones daily. Statistical significance was determined at the 0.05 level. The strength of associations was assessed using Cramer’s *V*.

All data were analysed using SPSS software (Version 28), ensuring reliable and accurate results. The analysis provided insights into the prevalence of PSU among university students, the role of smartphone usage patterns and any significant differences based on demographic factors.

### Measuring problematic smartphone use

The SAS-SV was utilised to assess the PSU levels of participants. The SAS-SV is a commonly used tool for measuring PSU.^10,11^ It consists of 10 items scored on a 6-point Likert scale, with a final score of 31 or higher indicating PSU.^12,13^

For each item, participants indicated their level of agreement using a 6-point Likert scale ranging from strongly disagree (1), disagree (2), partly disagree (3), partly agree (4), agree (5), to strongly agree (6). The SAS-SV assesses six addictive symptoms namely loss of control, disruption of family or schooling, disregard for consequences, withdrawal, preoccupation and tolerance.^14^ In the list below, the 10 items of the SAS-SV are indicated as they relate to each symptom:^12,14^

- **Loss of control:** I miss planned work due to smartphone use; I am constantly checking my smartphone so as not to miss conversations between other people on social media.
- **Disruption of family or schooling:** I am having a hard time concentrating in class, while doing assignments, or while working due to smartphone use; The people around me tell me that I use my smartphone too much.
- **Disregard for consequences:** I am feeling pain in my wrists or at the back of my neck while using a smartphone; I will never give up using my smartphone even when my daily life is already greatly affected by it.
- **Withdrawal:** I will not be able to tolerate not having a smartphone; I feel impatient and anxious when I am not holding my smartphone.
- **Preoccupation:** I have my smartphone in my mind even when I am not using it.
- **Tolerance:** I often use my smartphone longer than I had intended.

The original SAS-SV demonstrates high internal consistency (α = 0.92).^12^ For this study, the internal consistency was also high (α = 0.823). In this context, internal consistency refers to the extent to which all items within the questionnaire measure the same underlying concept. A higher Cronbach’s alpha (α) value (close to 1) indicates better internal consistency.

### Ethical considerations

This study adhered to ethical guidelines to protect the rights and well-being of participants. Ethical approval was granted by the Research Ethics Committee (Human) of Nelson Mandela University (ethics clearance reference number 0262), ensuring the study met all ethical standards. Informed consent was obtained from all participants, who were provided with information regarding the study’s purpose and procedures as well as their rights as participants.

The survey was anonymous and participants were assured that their responses would remain confidential and used solely for research purposes. The online questionnaire did not collect personally identifiable information, and all data were stored securely to maintain participant privacy. Participants were informed that their participation was voluntary and that they could withdraw from the study at any time without facing any consequences.

## Results

### Descriptive analysis

To determine the minimum sample size required to meet the desired statistical parameters, a 95% confidence level, 5% margin of error and a 50% population proportion were used. Based on the 31 311 registered students at the time of data collection, the minimum required sample size was calculated to be 380. A total of 867 students participated in the study.

Most participants were female (68%), while male participants accounted for 31% and 1% preferred not to disclose their sex. Regarding academic faculties, most participants were from the Faculty of Business and Economic Sciences (34%), followed by the Faculty of Engineering, the Built Environment, and Technology (17%). In addition, 13% of participants came from each of the Faculties of Humanities and Science, while 12% were from the Faculty of Health Sciences. The Faculty of Education represented 6%, and the Faculty of Law had the lowest number of participants, with 5%.

In terms of academic qualifications, most participants were enrolled in an Undergraduate Bachelor’s Degree programme, representing 53% of the sample, followed by those pursuing an Undergraduate Diploma (30%). Other qualifications had smaller representations: Undergraduate Higher Certificate (5%), Undergraduate Advanced Diploma (4%), and Postgraduate Master’s Degree (3%). Postgraduate Honours Degree and Postgraduate Diploma each accounted for 2%, with Postgraduate Doctoral Degree at 1%. Notably, no participants were registered for a Postgraduate Certificate.

Participants were asked about the amount of time they spend on their smartphones daily. The results showed that 45.1% of participants spent 8 h or more on their smartphones each day. Furthermore, 13.6% spent 6 h, 11.2% spent 5 h, 10.1% spent 4 h and 5.3% spent 3 h. Smaller percentages were reported for shorter durations: 2.8% spent 2 h, 1% spent 1 h and 0.9% spent less than 1 h daily. Only 0.3% of participants reported not using a smartphone at all.

**FIGURE 1:**
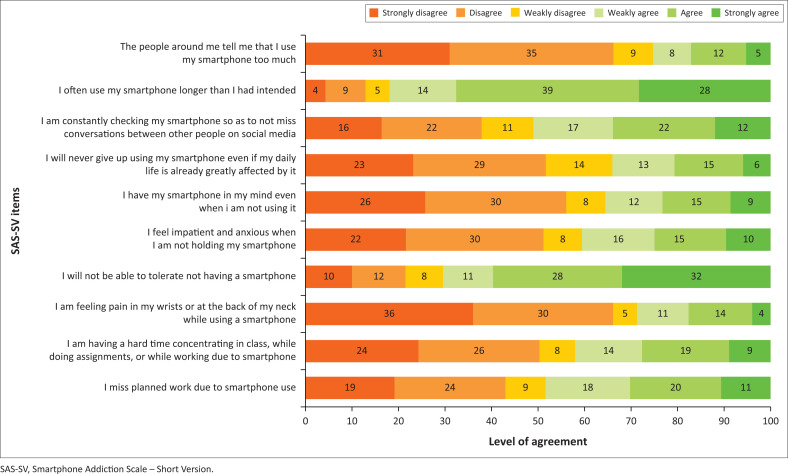
Response to Smartphone Addiction Scale – Short Version items.

Figure 1 indicates the responses to the 10 SAS-SV items that were included in the study. In the sections that follow, each of the SAS-SV items will be discussed as they relate to a PSU symptom.

### Loss of control

**FIGURE 2:**
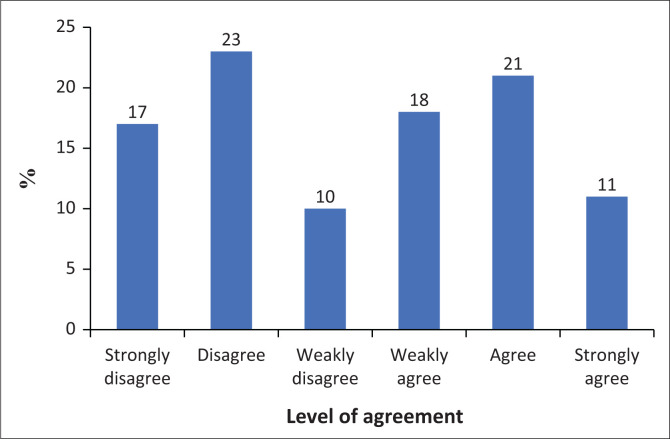
Loss of control results.

As shown in Figure 2, the results reveal a mixed response regarding the overall loss of control, presumably related to smartphone use. Twenty-three per cent of participants disagree with experiencing a loss of control, while 21% agree. In addition, 17% strongly disagree and 11% strongly agree. Eighteen per cent weakly agree, and 10% weakly disagree. Overall, there is a slight tendency towards agreement, with 50% of participants agreeing to some extent, compared to 50% disagreeing to some extent. This suggests that participants are evenly split on whether they experience an overall loss of control, indicating a variety of experiences among the respondents.

These results also show a division of opinions on whether participants miss planned work because of smartphone use. Twenty-three per cent disagree with the statement, while 20% agree. Nineteen per cent strongly disagree and 18% weakly agree. Eleven per cent strongly agree that smartphone use causes them to miss planned work, while 9% weakly disagree. The distribution across the agreement spectrum is relatively even, with a slight lean towards disagreement. The combined percentage of those who agree to some extent (49%) is slightly lower than those who disagree to some extent (51%), indicating a mixed impact of smartphone use on planned work, with a marginal tendency towards disagreement.

Lastly, responses regarding frequent smartphone checking for social media conversations are fairly evenly distributed. Twenty-two per cent of participants both disagree and agree with the statement, with 16% strongly disagreeing and 11% strongly agreeing. Seventeen per cent (17%) weakly agree, and 11% weakly disagree. Overall, there is a slight tendency towards agreement, with 50% of participants agreeing to some extent (weakly agree, agree, or strongly agree), compared to 49% disagreeing to some extent.

### Disruption of family or schooling

**FIGURE 3:**
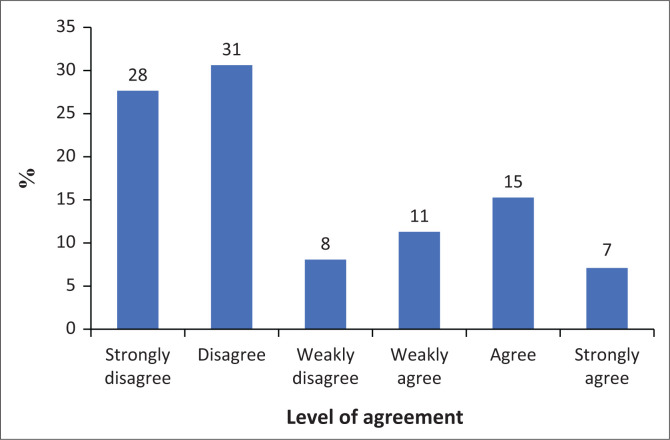
Disruption of family or schooling results.

Figure 3 shows that most participants tend to disagree with the statement that their smartphone disrupts their personal or academic lives. A total of 67% of participants disagree to some extent (strongly disagree, disagree, or weakly disagree), while 33% agree to some extent (strongly agree, agree, or weakly agree).

The results also indicate that the majority of participants reject the idea that others around them believe they use their smartphones excessively. Specifically, 36% disagree, and 31% strongly disagree with this claim. Only 12% agree, while 5% strongly agree. In addition, 9% weakly disagree and 8% weakly agree. Overall, 75% of participants disagree to some extent (strongly disagree, disagree, or weakly disagree), compared to 25% who agree to some extent. This suggests that most respondents do not feel they are criticised by others for excessive smartphone use.

Furthermore, there is a tendency among participants to disagree with the statement that smartphone use makes it difficult for them to concentrate in class, complete assignments, or work. A total of 24% strongly disagree, 26% disagree and 8% weakly disagree. In contrast, 42% agree to some extent (strongly agree, agree, or weakly agree), with 9% strongly agreeing, 19% agreeing and 14% weakly agreeing.

### Disregard for consequences

**FIGURE 4:**
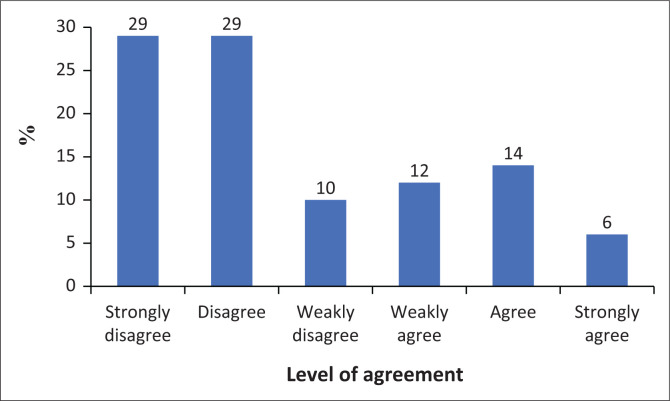
Disregard for consequences results.

The results in Figure 4 show that most participants disagree with experiencing overall disregard, likely related to smartphone use. Twenty-nine per cent strongly disagree, and 29% disagree with this idea. Fourteen per cent agree and 6% strongly agree. Twelve per cent (12%) weakly agree, and 10% weakly disagree. Overall, there is a clear tendency towards disagreement, with a combined 68% of participants disagreeing to some degree (strongly disagree, disagree, or weakly disagree), compared to 32% agreeing to some degree.

The results also indicate that most participants disagree with experiencing pain in their wrists or neck while using a smartphone. Thirty-six per cent (36%) strongly disagree, and 30% disagree with this statement. Fourteen per cent agree, while 4% strongly agree. Twelve per cent weakly agree, and 5% weakly disagree. Overall, there is a clear trend towards disagreement, with a combined 71% of participants disagreeing to some extent, compared to 29% agreeing to some extent.

Finally, the results show that most participants disagree with the statement that they would never give up using their smartphone, even if it significantly affected their daily lives. Twenty-nine per cent disagree, and 23% strongly disagree with this assertion. Fifteen per cent agree, 15% weakly disagree and 13% weakly agree. Only 5% strongly agree. Overall, there is a strong tendency towards disagreement, with a combined 67% of participants disagreeing to some extent (strongly disagree, disagree, or weakly disagree), compared to 33% agreeing to some extent. This suggests that most respondents would consider giving up their smartphone if it had a significant impact on their daily lives, although about a third of participants would be hesitant to do so.

### Withdrawal

**FIGURE 5:**
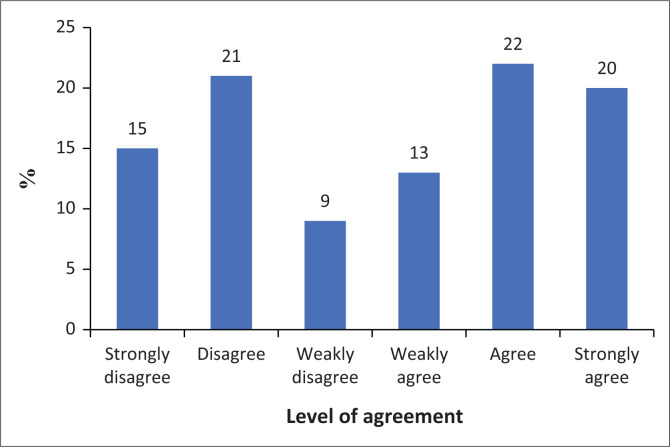
Withdrawal results.

Figure 5 illustrates a mixed response regarding overall withdrawal, presumably related to smartphone use. Twenty-two per cent of participants agree with experiencing withdrawal, while 21% disagree. Twenty per cent strongly agree, and 15% strongly disagree. Thirteen per cent weakly agree and 9% weakly disagree. Overall, there is a slight tendency towards agreement, with 55% of participants agreeing to some extent (weakly agree, agree, or strongly agree), compared to 45% disagreeing to some extent. This suggests that participants are somewhat divided on whether they experience withdrawal symptoms, with a slight majority reporting some level of withdrawal related to smartphone use.

The results also show a strong tendency towards agreement with the statement, ‘I won’t be able to tolerate not having a smartphone’. Thirty-one per cent of participants strongly agree, while 28% agree. Eleven per cent weakly agree with this statement. On the disagreement side, 12% disagree, 10% strongly disagree and 8% weakly disagree. Overall, a clear majority of participants agree, with 70% of participants agreeing to some extent (weakly agree, agree, or strongly agree), compared to 30% disagreeing to some extent. This suggests that a significant majority of respondents feel dependent on their smartphones and would struggle to live without one.

The results show that most participants disagree with the statement that they feel impatient and anxious when not holding their smartphone. Thirty per cent disagree and 21% strongly disagree with this statement. Sixteen per cent agree, while 9% strongly agree. Fifteen per cent weakly agree, and 9% weakly disagree. Overall, there is a clear tendency towards disagreement, with 60% of participants disagreeing to some extent (strongly disagree, disagree, or weakly disagree), compared to 40% agreeing to some extent. This indicates that most respondents do not experience significant impatience or anxiety when separated from their smartphones, although a notable minority does report such feelings.

### Preoccupation

**FIGURE 6:**
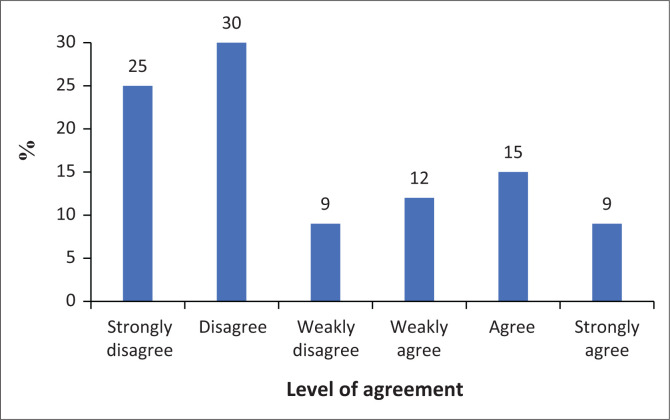
Preoccupation results.

The results show (see Figure 6) that most participants disagree with having their smartphone on their minds even when not using it. Thirty per cent (30%) disagree, and 25% strongly disagree with this statement. Fifteen per cent agree, while 8% strongly agree. Twelve per cent weakly agree and 9% weakly disagree. Overall, there is a clear tendency towards disagreement, with 64% of participants disagreeing to some extent, compared to 36% agreeing to some extent. This suggests that the majority of respondents do not often think about their smartphone when it’s not in use, although about a third of participants report some level of preoccupation with their device.

### Tolerance

**FIGURE 7:**
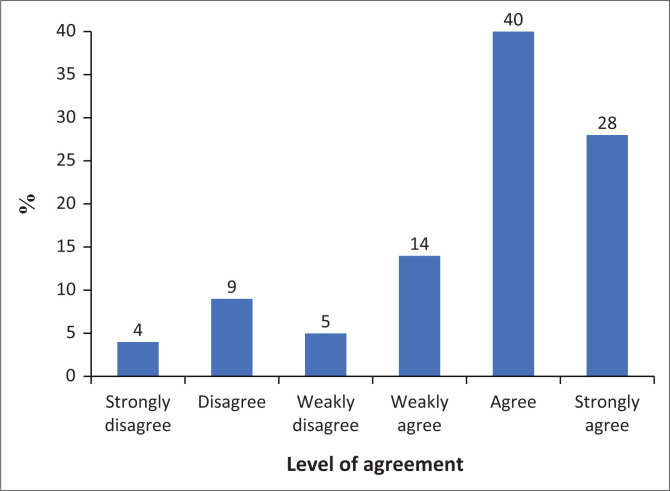
Tolerance results.

The results indicated in Figure 7 show a strong tendency towards agreement with the statement, ‘I often use my smartphone longer than I had intended’. Forty per cent (40%) of participants agree, while 28% strongly agree. Fourteen per cent weakly agree with the statement. On the disagreement side, 9% disagree, 5% weakly disagree, and 4% strongly disagree. Overall, there is a clear majority in agreement, with 82% of participants agreeing to some extent (weakly agree, agree, or strongly agree), compared to 18% disagreeing to some extent. This suggests that a large majority of respondents frequently use their smartphones for longer periods than initially planned, indicating a common tendency to exceed intended usage durations.

### Problematic smartphone use

To calculate the SAS-SV score, each of the 10 items is assigned a score based on the Likert scale response to the item.^12,13^ Scores range from 1 (strongly disagree), 2 (disagree), 3 (partly disagree), 4 (partly agree), 5 (agree), to 6 (strongly agree). A final score of 31 or higher indicates PSU.^12,13^ The maximum score that can be obtained is 60.

The mean SAS-SV score for participants is 32.4. Based on the results of this study, 55% (*n* = 477) of the participants could be classified as having problematic levels of smartphone use, also referred to as smartphone addiction. Previous research has shown that PSU is linked to various negative outcomes, including emotional disorders such as depression and anxiety,^15^ issues in interpersonal relationships,^2^ and even suicidal thoughts and behaviours.^16^ For students with PSU, there is also a decline in academic performance.^17^

### Hypothesis

The first hypotheses was as follows:

**H1:** There are significant differences in problematic smartphone use among university students based on gender.

The statistical analysis of PSU patterns among university students provides insightful findings regarding gender differences. The results of the chi-square test (χ^2^ = 16.34, *df* = 14, *p* = 0.293) and the Cramer’s *V* value of 0.097 suggest that there is no significant difference in PSU levels between male and female students.

Although there are noticeable variations – male students report higher percentages in the disagree (25%) and weakly disagree (40%) categories, while female students report slightly higher percentages in the weakly agree (30%) and agree (13%) categories – these differences are not statistically significant. This indicates that gender does not appear to be a significant factor in PSU among university students. Therefore, interventions or support programmes addressing PSU should likely be developed without focusing on gender-specific differences:

**H2:** There are significant differences in problematic smartphone use among university students based on the time they spend on their phones each day.

The thorough analysis of smartphone usage patterns and PSU levels among university students reveals a strong statistical relationship. The Chi-square test results (χ^2^ = 186.607, *df* = 63, *p* = 0.001) and Cramer’s *V* value of 0.175 indicate a significant correlation between students’ PSU levels and the amount of time they spend on their phones daily.

**FIGURE 8:**
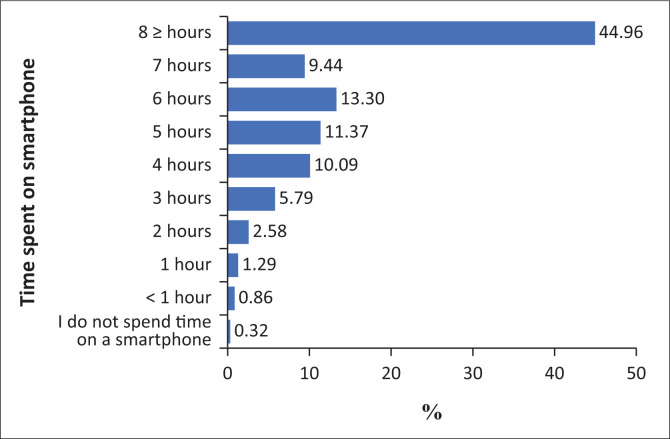
Responses related to time spent on smartphone.

The graph in Figure 8 clearly depicts this relationship, showing a distinct trend where students who spend eight or more hours on their smartphones are more likely to agree with PSU indicators, as evidenced by the taller bars in the agree and strongly agree categories. In contrast, those who report using their smartphones for fewer hours each day tend to strongly disagree with PSU-related statements. This pattern suggests that longer daily smartphone usage is associated with higher levels of self-reported PSU among university students.

However, it is important to acknowledge that while the relationship is statistically significant, Cramer’s *V* value of 0.175 suggests a moderate strength of association. This implies that while daily smartphone usage is an important factor in PSU, it is likely one of several factors influencing PSU patterns among university students.

## Discussion

The primary objective of this study was to examine the prevalence of PSU among university students at Nelson Mandela University in South Africa, with a focus on identifying potential gender differences and the impact of daily smartphone usage on PSU levels. The findings provide significant insights into the extent of PSU among students and highlight key factors that contribute to its prevalence.

### Problematic smartphone use

A striking result of this study is that 55% of participants were classified as having problematic levels of smartphone use, aligning with previous research that links PSU to negative outcomes such as emotional disorders, interpersonal relationship issues and reduced academic performance.^15,17^ The high prevalence of PSU in this population underscores the importance of addressing smartphone addiction in university settings, where students are often under significant academic and social pressures. Given that smartphone addiction is associated with numerous mental health challenges, the findings suggest that healthcare interventions in academic environments should prioritise promoting healthier smartphone habits.

When looking at international studies the SAS-SV scores vary across different studies and populations, reflecting the prevalence and severity of smartphone addiction among students. The mean SVS-SV score in this study was 32.4 and 55% of participants can be classified as a having problematic level of smartphone use. The SAS-SV scores among university and college students globally indicate a significant prevalence of smartphone addiction, as can be seen from the following international studies:

- **Turkey:** In a study at Ondokuz Mayis University, a significant portion of students had SAS-SV scores higher than the group mean, indicating notable smartphone addiction.^18^
- **Tunisia:** Among university students, the mean SAS-SV score was 38.3, with 75.9% considered at high risk of smartphone addiction.^19^
- **Saudi Arabia:** Medical students reported a mean SAS-SV score of 32.31, with 47.9% indicating smartphone addiction.^20^
- **China:** The prevalence of smartphone addiction among medical college students was 29.8%, with no significant gender differences.^21^
- **Serbia:** Among medical students, 19.5% were classified as addicted based on SAS-SV scores.^22^

### Problematic smartphone use according to gender

While the statistical analysis of gender differences in this study shows no statistically significant difference between PSU levels of male and female students, other research has found that gender differences in PSU reflect true differences rather than measurement biases.^23^ One such difference is the purpose the smartphone is used for. It has been found that PSU in male students is associated with gaming applications, anxiety and poor sleep quality.^20,24^ For female students, it is linked to multimedia applications, social media, depression, anxiety and poor sleep quality.^21,24^ This study’s findings suggest that gender is not a significant factor in determining PSU levels among university students. This implies that interventions aimed at reducing PSU could be more effective if they are designed to be gender-neutral, focusing on the overall addictive nature of smartphone use rather than tailoring strategies based on gender. The first hypothesis, which posited that there is a difference in smartphone use based on gender, is thus not supported by the results of this study.

### Problematic smartphone use based on smartphone usage

The second hypothesis, which posited that the amount of time spent on smartphones each day would correlate with PSU, was strongly supported by the data. A significant relationship was found between the daily duration of smartphone use and self-reported PSU levels, with students who spent eight or more hours on their smartphones being more likely to exhibit signs of addiction. This aligns with existing literature that suggests prolonged smartphone use is a key predictor of PSU, as extended screen time can lead to compulsive usage patterns that interfere with other aspects of daily life, such as academics, sleep and social relationships.^4,5^ Studies show that excessive smartphone use, often exceeding several hours daily, is linked to behaviours that meet the criteria for addiction, such as preoccupation, overuse, and negative impacts on daily life.^24,25^ This is particularly true when usage exceeds 4 h daily.^25,26^ In the current study, 89% of participants use their smartphones for 4 h or more. Excessive smartphone use is associated with stress, anxiety, sleep disorders and decreased productivity, impacting both psychological and physical health.^26,27,28^

The moderate strength of the association (Cramer’s *V* = 0.175) suggests that while time spent on smartphones is a significant factor in predicting PSU, it is not the sole determinant. This indicates that other factors, such as personality traits, academic stress, or social media engagement, might also contribute to the development of smartphone addiction. Future research should explore these additional variables to gain a more comprehensive understanding of the factors that contribute to PSU among university students.

### Problematic smartphone use symptoms

The study’s findings also shed light on the specific symptoms of PSU experienced by university students. Responses to the SAS-SV items revealed that a significant portion of students reported tendencies towards ‘tolerance’ (82% agreeing to some extent that they use their smartphones longer than intended) and ‘withdrawal’ (70% of students agreeing that they would struggle to live without their smartphones). These symptoms are commonly associated with addiction, suggesting that smartphone use among students may be reaching problematic levels. However, other symptoms such as ‘disruption of family or schooling’ and ‘disregard for consequences’ were less frequently reported, with most students indicating that their smartphone use did not interfere significantly with their academic or personal lives. This finding implies that while students may be exhibiting addictive behaviours, the broader consequences of PSU may not yet be as severe as in populations with more extreme cases of addiction.

Interestingly, the symptom of ‘preoccupation’ (thinking about one’s smartphone even when not using it) was less frequently reported, with 64% of participants disagreeing to some extent with the statement. This suggests that while students may feel compelled to use their smartphones for extended periods, the constant preoccupation with the device may not be as pervasive as other addiction-related behaviours.

### Implications for interventions

The findings from this study have important implications for developing interventions to address PSU among university students. Given the high prevalence of PSU, particularly among those who use their smartphones for eight or more hours daily, universities should consider implementing strategies to encourage healthier smartphone usage. Interventions could include educational campaigns about the risks of excessive smartphone use, as well as programmes aimed at promoting time management and self-regulation skills.

Additionally, healthcare providers and university counsellors should be trained to identify the signs of PSU early, especially given its potential links to mental health issues such as anxiety and depression. Counselling services could incorporate strategies to help students manage their smartphone use, focusing on improving digital well-being and promoting mindfulness techniques that encourage students to engage more fully in academic and social activities offline.

### Contribution

The following four points highlight the contribution of this article:

- **Potential for early intervention:** Primary care settings offer an opportunity to intervene early in cases of PSU, potentially preventing more severe consequences. By promoting healthier digital habits and offering counselling or resources for managing smartphone use healthcare professionals can support students in achieving better mental health outcomes and enhancing their quality of life.
- **Prevalence of PSU:** The study found that 55% of university students at Nelson Mandela University exhibited PSU, highlighting the widespread nature of smartphone addiction among students.
- **Association with daily usage time:** A significant correlation was found between the amount of time students spend on their smartphones each day and the severity of PSU, with longer daily usage leading to higher levels of addiction symptoms.
- **No gender differences in PSU:** The study revealed no significant gender differences in PSU, suggesting that interventions should focus on reducing smartphone usage duration rather than targeting specific genders.

### Limitations

While this study provides valuable insights into PSU among university students, it has some limitations. Firstly, the cross-sectional design of the study meant that causality could not be established. Longitudinal studies would provide a better understanding of how smartphone usage patterns and PSU evolve over time and how they may impact academic success and mental health. Secondly, the study relied on self-report measures, which might have been subject to social desirability bias or inaccurate reporting. Future research could incorporate more objective measures, such as smartphone usage tracking, to complement self-reported data.

Furthermore, the study focused on students at a single university in South Africa, which might have limited the generalisability of the findings to other contexts. It would be beneficial for future studies to replicate this research in different cultural and geographical settings to assess the universality of the findings and to explore cultural factors that may influence smartphone usage patterns.

## Conclusion

This study found a significant link between daily smartphone use and PSU among university students, with those using their phones for eight or more hours showing higher PSU levels. No gender differences in PSU were observed. With 55% of students exhibiting problematic use, this underscores the need for primary healthcare interventions to address the mental health and academic impacts of excessive screen time. These findings highlight the importance of integrating strategies to manage smartphone use into primary healthcare, especially in university settings, to support students’ well-being.
